# *Alicyclobacillus mali* FL18 as a Novel Source of Glycosyl Hydrolases: Characterization of a New Thermophilic β-Xylosidase Tolerant to Monosaccharides

**DOI:** 10.3390/ijms232214310

**Published:** 2022-11-18

**Authors:** Flora Salzano, Martina Aulitto, Gabriella Fiorentino, Emilia Pedone, Patrizia Contursi, Danila Limauro

**Affiliations:** 1 Department of Biology, University of Naples Federico II, Via Cinthia, 80126 Naples, Italy; 2 Division of Biological Systems and Engineering, Lawrence Berkeley National Laboratory, Berkeley, CA 94720, USA; 3 Institute Biostructures and Bioimaging, National Research Council, Via Pietro Castellino 111, 80131 Naples, Italy

**Keywords:** β-xylosidase, thermophiles, glycosyl hydrolases, *Alicyclobacillus mali*

## Abstract

A thermo-acidophilic bacterium, *Alicyclobacillus mali* FL18, was isolated from a hot spring of Pisciarelli, near Naples, Italy; following genome analysis, a novel putative β-xylosidase, AmβXyl, belonging to the glycosyl hydrolase (GH) family 3 was identified. A synthetic gene was produced, cloned in pET-30a(+), and expressed in *Escherichia coli* BL21 (DE3) RIL. The purified recombinant protein, which showed a dimeric structure, had optimal catalytic activity at 80 °C and pH 5.6, exhibiting 60% of its activity after 2 h at 50 °C and displaying high stability (more than 80%) at pH 5.0–8.0 after 16 h. AmβXyl is mainly active on both para-nitrophenyl-β-D-xylopyranoside (K_M_ 0.52 mM, *k*_cat_ 1606 s^−1^, and *k*_cat_/K_M_ 3088.46 mM^−1^·s^−1^) and para-nitrophenyl-α-L-arabinofuranoside (K_M_ 10.56 mM, *k*_cat_ 2395.8 s^−1^, and *k*_cat_/K_M_ 226.87 mM^−1^·s^−1^). Thin-layer chromatography showed its ability to convert xylooligomers (xylobiose and xylotriose) into xylose, confirming that AmβXyl is a true β-xylosidase. Furthermore, no inhibitory effect on enzymatic activity by metal ions, detergents, or EDTA was observed except for 5 mM Cu^2+^. AmβXyl showed an excellent tolerance to organic solvents; in particular, the enzyme increased its activity at high concentrations (30%) of organic solvents such as ethanol, methanol, and DMSO. Lastly, the enzyme showed not only a good tolerance to inhibition by xylose, arabinose, and glucose, but was activated by 0.75 M xylose and up to 1.5 M by both arabinose and glucose. The high tolerance to organic solvents and monosaccharides together with other characteristics reported above suggests that AmβXyl may have several applications in many industrial fields.

## 1. Introduction

Efficient utilization and valorization of lignocellulosic biomass are important in the shift to a biobased society in which biochemical processes are regarded as the most sustainable alternatives to physicochemical treatments [1]. Lignocellulose, an organic fibrous material obtained from natural wood and agro-industrial residues, is composed mainly of cellulose, hemicellulose, and lignin [2]. Xylan is the main component of hemicellulose, and it consists of β-(1,4)-linked D-xylopyranose residues that can be decorated with α-L-arabinofuranose, 4-O-D-methyl-glucuronic acids, and acetyl groups in nature [3]. To convert xylan into its constituent sugars, an array of enzymatic activities including endoxylanase (EC 3.2.1.8) and β-xylosidase (EC 3.2.1.37) together with α-glucuronidase (E.C.3.2.1.139), α-arabinofuranosidase (E.C.3.2.1.55), and acetylxylan esterase (E.C.3.1.1.72) is required. Endoxylanase randomly cleaves the internal β-(1,4) linkages of xylan to yield xylooligosaccharides (XOS) [4]; β-xylosidase, which is an exoglycosidase, removes β-xylosyl residues from non-reducing ends of xylobiose and XOS, being essential to relieve the end-product inhibition of endoxylanase during complete hydrolysis of xylan. However, a synergistic action of the other enzymes is required to completely degrade the polymer [5].

β-xylosidases are classified into the glycoside hydrolase (GH) families 3, 5, 30, 39, 43, 51, 52, and 120 (http://www.cazy.org/Glycoside-Hydrolases.html, accessed on 15 May 2022) according to the amino-acid sequence similarities. They operate through a mechanism of inversion (GH family 43) or retention (GH families 3, 30, 39, 43, 51, 52, and 120) of the stereochemical configuration at the anomeric carbon [6]. β-Xylosidases have found application in a wide range of industrial processes, such as bleaching of paper pulp, enhancing the digestibility and nutritional properties of animal feed, manufacturing of beer and wine, clarification of fruit juices, and extraction of coffee [7]. They are also commonly used in biorefinery processes during the saccharification of pretreated agro-industrial wastes to produce biofuels [8,9,10,11,12]. Even now, significant efforts are being made to isolate and characterize increasingly high-performance and resistant enzymes for efficient biomass utilization. Thermophilic and thermostable glycosyl hydrolases from thermophilic microorganisms are generally used to promote faster reactions, increase substrate solubility, and reduce the risk of contamination [13,14,15,16]. However, most of the β-xylosidases are known to be inhibited by the hydrolysis products. Considering that, during the saccharification processes, high concentrations of monosaccharides are released, the research of new tolerant GHs is needed to increase the efficiency of substrate hydrolysis and lower the cost of many industrial processes [4,17,18,19,20].

In the present work, we performed cloning and heterologous expression of a putative β-xylosidase (AmβXyl) based on the most recent genomic annotation of *Alicyclobacillus mali* FL18, an interesting thermo-acidophilic microorganism isolated from a hydrothermal hot spring of Pisciarelli, near Naples, in Italy [21]. Additionally, we purified the recombinant enzyme and exhaustively investigated the sequence identity, enzymatic properties, xylose tolerance, and activity against natural XOS new monosaccharides/solvent-tolerant enzymes that improve the saccharification of pretreated lignocellulosic biomass into fermentable sugars, e.g., for the production of value-added products. 

## 2. Results

### 2.1. Sequence Analysis

According to the analysis of the genome sequence of *A. mali* FL18 (accession No. JADPKZ0000000) [21], the 2360 bp gene encoding putative 1,4 β-xylosidase (AmβXyl) (Accession No. MBF8378422.1) of 783 amino acids (MW 84.70 kDa and pI 5.13) was highlighted. The putative protein belongs to the GH3 family, a heterogeneous family that includes various GH activities such as β-glucosidase, xylan 1,4-β-xylosidase, β-glucosylceramidase, β-N-acetyl-hexosaminidase, α-L-arabinofuranosidase, glucan 1,4-β-glucosidase, exo-1,3-1,4-glucanase, β-N-acetylglucosaminide phosphorylase, β-1,2-glucosidase, β-1,3-glucosidase, xyloglucan-specific hexo-β-1,4-glucanase/hexo-xyloglucanase, and lichenase/endo-β-1,3-1,4-glucanase. In addition, genome analysis showed two putative ATP-binding cassette (ABC) permeases upstream of the AmβXyl gene (accession no. WP_230088200.1 and WP_230088182.1), which could be used for the uptake of sugars [22,23].

To express the recombinant protein in *E. coli*, SignalP 6.0 and NetNGlyc bioinformatics analyses were performed. The results showed the absence of a signal peptide and the presence of a single N-glycosylation site (Asn518), conditions that could allow the expression in *E. coli* as a soluble protein.

**Figure 1:**
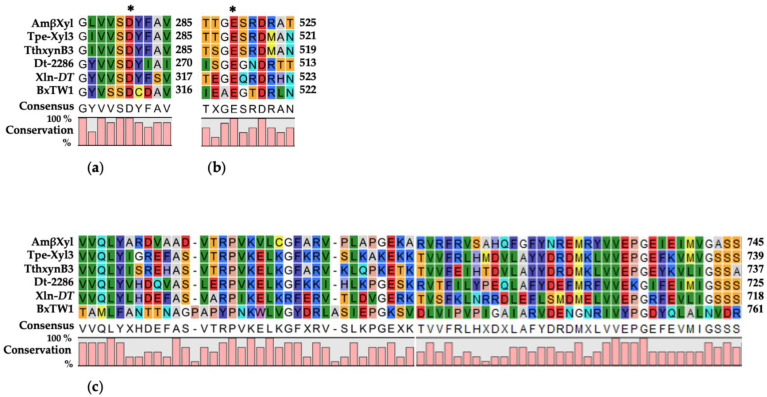
Sequence homology of β-xylosidases from different sources. The amino-acid sequences of *A. mali* FL18, *T. petrophila*, *P. thermarum, D. turgidum*, *D. thermophilum*, and *T. amestolkiae* were aligned to optimize the sequence similarity. (**a**,**b**) Amino-acid stretches surrounding the catalytic nucleophile (Asp281) and the general acid/base (Glu519) residues, respectively, involved in the catalysis; these residues are marked with an asterisk. (**c**) Fibronectin type III-like domain. Colors code is automatically generated by CLC Main Workbench in according to the amino-acid residues.

Multiple sequence alignment and BlastP analyses indicated homology of AmβXyl with other thermophilic β-xylosidases from *Thermotoga petrophila* (accession No. ABQ46867.1)*, Pseudothermotoga thermarum* (accession No. AEH50242.1)*, Dictyoglomus turgidum* (accession No. ACK42133.1)*,* and *D. thermophilum* (accession No. WP012548714.1), with 54%, 51%, 50%, and 43% identity, respectively. In addition, I-TASSER and Pfam analyses highlighted some typical structural features of GH3 family: the presence of the amino-acid sequences of two conserved residues, Asp281 (nucleophile) and Glu519 (acid/base), involved in the retaining reaction mechanism (Figure 1a,b) and the fibronectin type III-like domain (Figure 1c), which is generally found at the C-terminus of enzymes belonging to the GH3 family [7].

**Figure 2:**
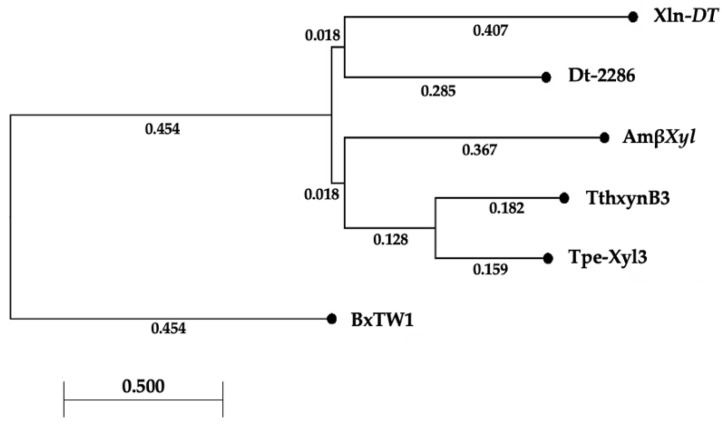
Phylogenetic analysis built on the basis of the multiple alignment of amino-acid sequences (numbers on nodes correspond to percentage bootstrap values for 200 replicates).

To investigate the evolutionary relationship among thermophilic GH3 β-xylosidases, the phylogenetic tree was constructed using the CLC Main Workbench 22.0.1 tool with the neighbor-joining (NJ) method (Figure 2).

The tree showed that AmβXyl has a distant relationship from *P. thermarum* and *T. petrophila*, despite a high sequence identity, suggesting possible different enzymatic features. 

### 2.2. Purification and Quaternary Structure of AmβXyl 

**Figure 3:**
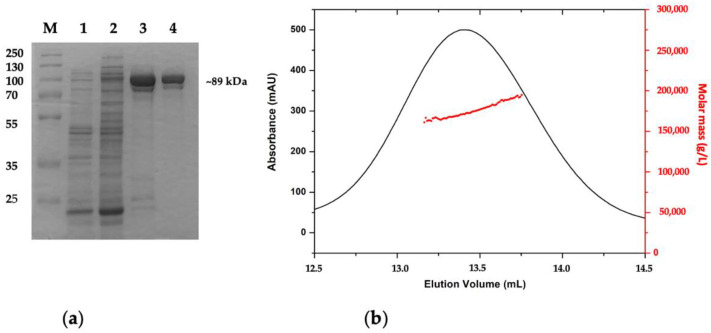
Analysis of the recombinant AmβXyl expressed in *E. coli* BL21(DE)RIL/pET30a-AmβXyl. (**a**) SDS-PAGE of the purification steps. M, molecular mass markers. (1) C.E. from non-induced cells; (2) C.E. from IPTG-induced cells; (3) A.C.; (4) A.E.C. (**b**) Analysis of purified AmβXyl by gel filtration chromatography coupled with light scattering QELS. The elution profile of AmβXyl is shown as a continuous line. The clustered points represent the light scattering data converted to molecular mass.

The AmβXyl gene was synthetically produced and codon-adapted to *Escherichia coli* genetic system. The gene was cloned in pET-30a(+) vector and it was expressed in *E. coli* BL21 (DE3) RIL. The recombinant protein was purified, almost to homogeneity, by His-trap affinity chromatography and anionic exchange chromatography, with a final amount of 2 mg for 1 L of culture. SDS-PAGE analysis showed a single-band with a molecular mass of ~89 kDa, which is in line with the predicted molecular weight (Figure 3a). The purification steps were summarized in Table 1, which shows a yield and purification ratio of 12% and 26.80 times, respectively, after anionic exchange chromatography.

To gain insight into the quaternary structure, purified AmβXyl was analyzed by size-exclusion chromatography coupled with a triple-angle light scattering QELS. The result (Figure 3b) showed a molecular weight of about 177 kDa ± 0.1% (Rh = 5.7 nm ± 2%), indicative of a dimeric structure of AmβXyl in solution. Within the prokaryotes, many β-xylosidases belonging to GH3 family are characterized, but few quaternary structures are known [7]; among these, β-xylosidase from *Streptomyces* CH7 adopts a dimeric structure, while β-xylosidase from *Streptomyces thermoviolaceus* OPC-520 is monomeric [24,25].

### 2.3. Characterization of AmβXyl 

#### 2.3.1. Biochemical Properties 

The influence of temperature and pH to determine the optimal activity and stability of AmβXyl was tested on PNP-β-xyl as substrate. Enzymes that work in a wide range of pH and high-temperature values are desirable in biotechnology for various applications, such as acid and alkaline pretreatment or steam explosion to generate bioethanol from plant biomass, such as wheat straw [26].

**Figure 4:**
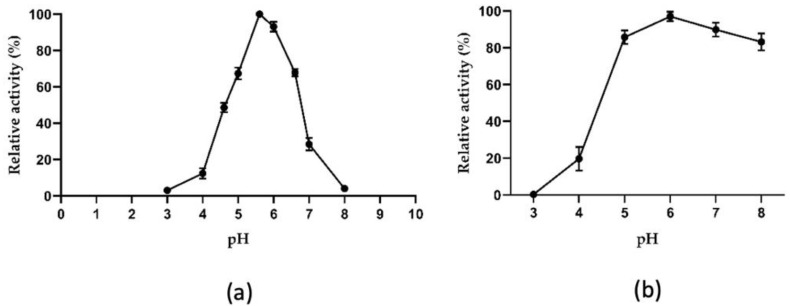
Effect of pH on the enzymatic activity of AmβXyl. (**a**) The pH optimum was evaluated in buffers ranging from pH 3.0 to pH 8.0. (**b**) The pH stability was determined by incubating AmβXyl in various buffers (from pH 3.0 to pH 8.0) for 16 h and assaying residual activity under optimal conditions. The activity of AmβXyl was determined using PNP-β-xyl as substrate. The concentration of released *p*NP was detected by measuring A_405nm_.

AmβXyl was tested in the range of pH values from 3.0 to 8.0, showing an optimal pH at 5.6 and retaining over 70% activity in a range of pH from 5.0 to 6.6 and ~50% activity at pH 4.5 (Figure 4a).

A similar trend in pH profile was observed also for the other thermophilic GH3 β-xylosidases, such as Dt-xyl3 from *D. turgidum*, Dt-2286 from *D. thermophilum*, Tpe-Xyl3 from *T. petrophila*, and TthxynB3 from *T. thermarum*, which exhibit optimal activity in the pH range from 5.0 to 6.0. Approximately 70% of the maximum activity was retained in the pH ranging from 5.0 to 7.0 for these enzymes [27,28,29,30]. As shown in Figure 4b, the enzyme was stable in a wide pH range after 16 h of incubation at 4 °C in the various buffers, retaining 95% activity at pH 6.0 and 80% activity in the pH range from 5.0 to 8.0 in line with β-xylosidase from the thermophilic bacterium *C. crescentus,* which has similar pH activity ranges with an optimum close to 6.0 and stability between 5.0 and 10.0 at 4 °C for 24 h [31]. This remarkable pH stability points to AmβXyl as suitable for hydrolyzing acid or alkaline pretreated lignocellulose. 

**Figure 5:**
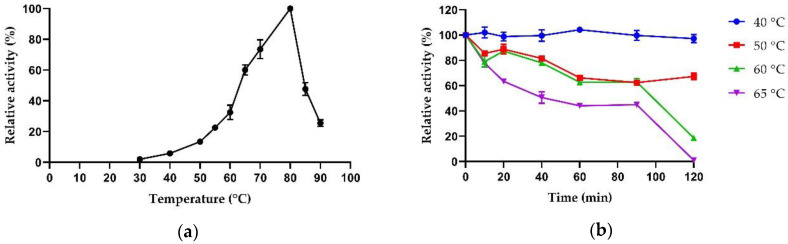
Effect of temperature on the enzymatic activity of AmβXyl. (**a**) The optimum temperature was determined by measuring enzyme activity in the range 30–90 °C. (**b**) Thermostability was determined by incubating the enzyme at 40 °C, 50 °C, 60 °C, and 65 °C for different times and then assaying for residual activity under optimal conditions. The activity of AmβXyl was determined using PNP-β-xyl as a substrate. The concentration of released *p*NP was detected by measuring A_405nm_.

Activity and stability of catalysts at high temperatures are also properties worthy of attention in industrial processes for various reasons; high temperatures reduce the risk of microbial contamination, increase the solubility of substrates, and reduce the cost of cooling the industrial biorefinery plant, allowing the volatilization of products such as ethanol [32]. Many β-xylosidases, already characterized in the literature, isolated from both fungi and thermophilic bacteria, show a temperature optimum in the range of 50–65 °C, such as PtXyl43 from the fungus *Paecilomyces thermophila* [33] or TtGH39 from the bacterium *Thermoanaerobacterium thermosaccharolyticum* [34]. Only one β-xylosidase from *Alicyclobacillus* genus has been reported with an optimal temperature of 65 °C [35]. AmβXyl showed a temperature optimum of 80 °C and it also retained more than 50% activity between 65 °C and 85 °C, while a decrease in activity was observed at lower temperatures and at 90 °C (Figure 5a).

Therefore, AmβXyl is one of the most thermophilic β-xylosidases characterized so far, together with those from *T. petrophila* (90 °C), *T. thermarum* (95 °C) and *D. turgidum* (98 °C) [27,29,30].

The thermal inactivation of the enzyme was also examined; it was stable for 120 min at 40 °C and retained more than 50% activity up to 120 min at 50 °C and 60 min at 60 °C and 65 °C (Figure 5b), whereas there was no activity after 5 min at 70 °C and 80 °C. AmβXyl was less thermostable than the homologous enzymes of *T. thermarum* and *D. turgidum*, but more thermostable compared to other characterized β-xylosidases, e.g., β-xylosidase from *Aspergillus niger* which is completely inactive after only 40 min of incubation at 65 °C [36], and β-xylosidase from *Enterobacter ludwigii*, which loses completely activity after 20 min at 55 °C [37].

#### 2.3.2. Substrate Specificity

Both β-linked and α-linked artificial glycoside substrates and natural substrates were used to analyze the specificity of AmβXyl. The enzyme showed higher specific activity toward PNP-β-xyl than PNP-α-ara (Table 2). This bifunctional enzymatic activity, which might be correlated to the spatial similarity between D-xylopyranose and L-arabinofuranose [38], is present mainly in families 3, 43, and 53 [7], and it was found in the homologous GH3 β-xylosidases from *T. petrophila* (TpeXyl3) [30] and *T. thermarum* (TthxynB3) [29]. Enzymatic activity was measured on other synthetic substrates; slight activity was observed on PNP-α-glu (0.19%) and PNP-β-glu (0.73%), while no activity was reported toward PNP-α-gal and PNP-β-gal (Table 2) in comparison to PNP-β-xyl. Furthermore, AmβXyl was weakly active on the natural substrates beechwood xylan and wheat arabinoxylan (14.59% and 20.86%, respectively), similar to Xyl43A and Xyl43B from *Humicola insolens* [39].

**Figure 6:**
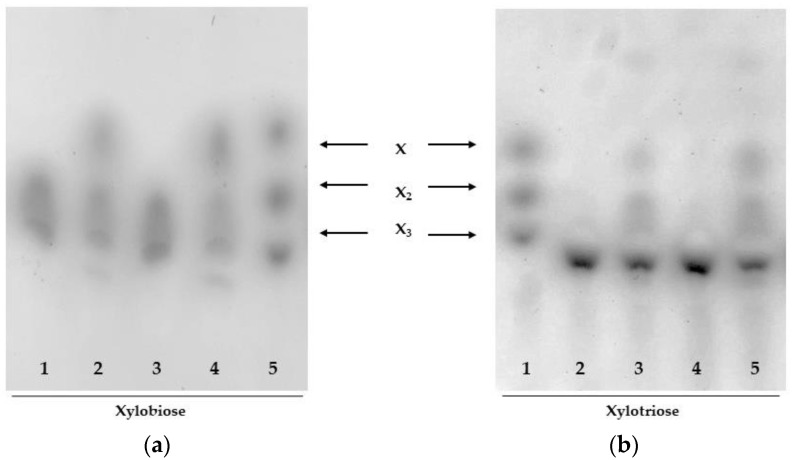
TLC analysis of xylobiose and xylotriose hydrolyzed by AmβXyl. (**a**) Xylobiose hydrolysis: (1) control reaction without enzyme (1 min); (2) hydrolysis products (1 min); (3) control reaction without enzyme (30 min); (4) hydrolysis products (30 min); (5) standards XOS (X, X_2_, X_3_). (**b**) Xylotriose hydrolysis: (1) standards XOS (X, X_2_, X_3_); (2) control reaction without enzyme (1 min); (3) hydrolysis products (1 min); (4) control reaction without enzyme (30 min); (5) hydrolysis products (30 min).

An important feature of β-xylosidases, for biotechnological application, is their ability to hydrolyze natural substrates such as XOS, since they are often found in hydrolysates of lignocellulosic material and are potent inhibitors of endo-β-1,4-xylanases and cellulases [7,40,41]. Therefore, xylobiose and xylotriose were incubated for different times with purified AmβXyl and examined by TLC (Figure 6). A thermal effect on the migration of xylobiose (Figure 6a, lanes 1 and 3) and xylotriose (Figure 6b, lanes 2 and 4) was observed at the assay temperature; however, it did not influence XOS hydrolysis. Results showed that the enzyme hydrolyzed these short XOS into xylose (X), demonstrating its true xylosidase activity. Interestingly, the enzyme hydrolyzed X_2_ in X (Figure 6a) and X_3_ in X_2_/X after only 1 min of incubation (Figure 6b). This capability of hydrolyzing XOS makes AmβXyl a crucial enzyme to improve the efficiency of the saccharification process.

#### 2.3.3. Kinetic Parameters

The kinetic parameters of AmβXyl were measured for both PNP-β-xyl and PNP-α-ara at optimal pH and temperature (Table 3).

AmβXyl showed higher activity on PNP-β-xyl than PNP-β-ara with K_M_, *k_cat_*, and *k_cat_*/K_M_ values of 0.52 mM, 1606.00 s^−1^, 3088.46 mM^−1^·s^−1^ respectively, while kinetic parameters for AmβXyl on PNP-α-ara were K_M_ = 10.56 mM, *k*_cat_ = 2395.80 s^−1^, and *k*_cat_/K_M_ = 226.87 mM^−1^·s^−1^.

As illustrated in Table 3, the comparison of kinetic parameters with other thermophilic GH3 β-xylosidases highlighted that AmβXyl exhibited a lower catalytic efficiency for PNP-β-xyl (~2.5-fold) than TthxynB3 from *T. petrophila* but a higher catalytic efficiency for PNP-β-xyl (~2.5-fold) than TthxynB3 from *T. thermarum*. Regarding the enzymatic activity toward PNP-β-ara, only the catalytic efficiency of Dt-2286 from *D. turgidum* showed a 10-fold increase with respect to AmβXyl; the other β-xylosidases reported in Table 3 showed similar catalytic efficiency.

#### 2.3.4. Effect of Chemical Agents

Metal ions can be liberated during biomass degradation as a consequence of corrosion of the equipment used, leading to enzyme inhibition [42]. Recombinant AmβXyl shows good tolerance to metals in accordance with the characteristics of *A. mali* FL18 strain, which is particularly tolerant to nickel, cobalt, mercury, and other metals, having been isolated from an arsenic-rich hot spring [21]. As reported in Table 4, metal ions and EDTA at the final concentrations of 1 and 5 mM did not inhibit the enzyme activity, which remained between 80% and 100%, except for Cu^2+^ at the concentration of 5 mM, which strongly inhibited AmβXyl activity.

A similar effect of Cu^2+^ was observed in both enzymes Dt-2286 and Dt-Xyl3 from *D. turgidum* [27,28]. Cu^2+^ ion is known to catalyze the auto-oxidation of cysteine residues, leading to the formation of intra- and intermolecular disulfide bonds or sulfenic acid [28]. Indeed, eight cysteine residues are present in the AmβXyl amino-acid sequence, and their oxidation state might influence the structure/function of the enzyme. Furthermore, at different concentrations of EDTA, no significant effect was observed, suggesting that this chelating agent did not affect enzymatic activity.

Regarding the effect of surfactants, AmβXyl was only found to be very sensitive to the anionic detergent SDS (Table 4). Conversely, the nonionic detergents Tween-20 and Triton X-100 did not inhibit enzyme activity. These surfactants are often used during bioconversion of lignocellulosic biomass as they can bind to the residual lignin of biomass through hydrophobic interactions, thus preventing the adsorption of hydrolytic enzymes on lignin, and they are also able to protect enzymes from denaturing by heat [43,44].

**Figure 7:**
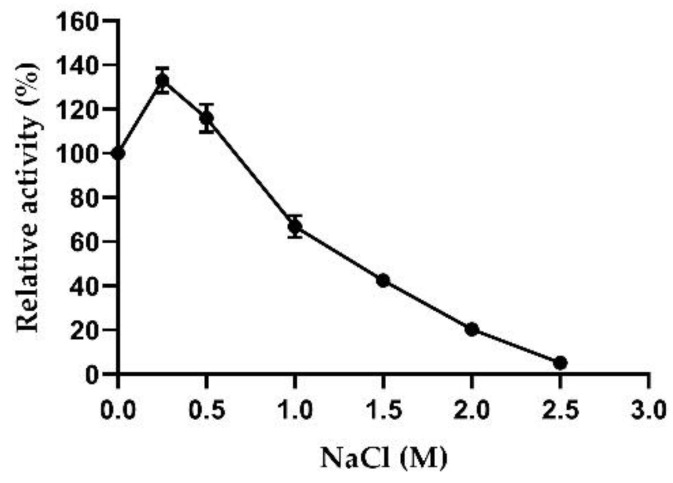
Effect of NaCl on AmβXyl activity.

Halotolerance is an interesting feature of xylanases and xylosidases for applications in the treatment of saline foods, such as soy sauce, which have a salt concentration between 0.5 and 2.5 M [45,46,47]. For this reason, AmβXyl activity was evaluated with increasing NaCl concentrations. As shown in Figure 7, the enzyme activity gradually decreased with increasing NaCl concentrations, maintaining more than 100% activity at 0.5 M NaCl and approximately 50% activity in presence of 1 M NaCl, suggesting the possible use of AmβXyl in saline food processing.

**Figure 8:**
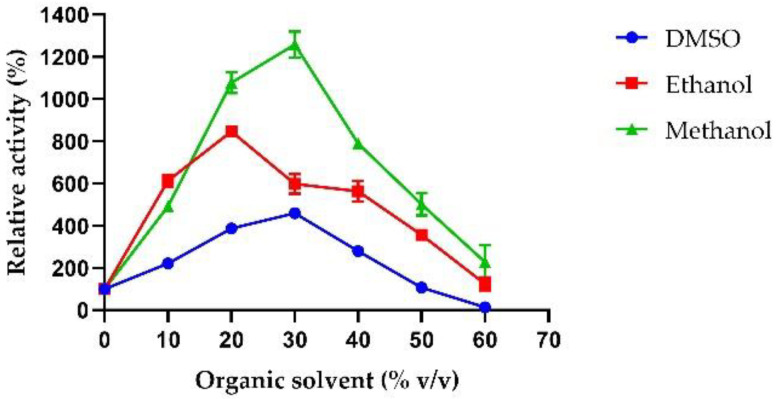
Effect of organic solvents on AmβXyl activity. DMSO (circles), ethanol (squares), and methanol (triangles).

Lastly, organic solvents are often used during biomass saccharification to improve the solubilization of water-insoluble substrates and to overcome biomass recalcitrance to hydrolysis [48]. Therefore, the effect of DMSO, ethanol, and methanol on AmβXyl activity at different concentrations was analyzed. As shown in Figure 8, AmβXyl was active at high concentrations of solvents; enzymatic activity increased about fourfold in the presence of 30% DMSO, ninefold in the presence of 20% ethanol, and 12-fold in the presence of 30% methanol. Only in the presence of 60% DMSO was a slight reduction in activity compared with the control determined. This effect could be attributed to the presence of a higher percentage of hydrophobic amino acids in the thermostable enzymes compared to their mesophilic counterparts [49]. Indeed, AmβXyl possesses 383 hydrophobic amino-acid residues (~46%) that might contribute to its high tolerance toward organic solvents. Organic solvent tolerance is not frequently reported in other thermophilic β-xylosidases; for example, TpeXyl3 from *T. petrophila* shows a slight increase in enzymatic activity in the presence of 15% of methanol while the activity is reduced by 50% in the presence of DMSO or methanol at the concentration of 30% [30].

This result suggests that AmβXyl is an enzyme that can efficiently work in the presence of an organic solvent that is required for the biotransformation of a poorly water-soluble substrate and that the tolerance of organic solvents makes AmβXyl a suitable enzyme to be used to clarify alcoholic beverages such as wine or beer.

#### 2.3.5. Sugar Tolerance of AmβXyl 

Xylose is a strong inhibitor of β-xylosidases; therefore, xylose-tolerant enzymes have great potential in biotechnological applications. In addition, the inhibition of enzymatic activity by glucose or arabinose of β-xylosidases is also a serious bottleneck in industrial biomass saccharification since these monosaccharides may accumulate to high concentrations reducing the enzymatic activity [7]. Hence, monosaccharide-tolerant β-xylosidases are of great interest; however, unfortunately, previous studies have underlined that most β-xylosidases are generally sensitive to the inhibition at low concentrations of these sugars. β-Xylosidase from *Trichoderma harzianum* C completely loses activity at 2 mM xylose [50], while β-xylosidase from *H. insolens* is 50% inhibited by 29 mM xylose [51]. Among thermophilic GH3 β-xylosidases, the enzyme from *T. thermarum* loses 50% activity in the presence of 1 M xylose [29], while Dt-2286 from *D. turgidum* shows tolerance until 1 M arabinose and a strong decrease in enzymatic activity in the presence of both xylose and glucose by 0.5 M [27].

**Figure 9:**
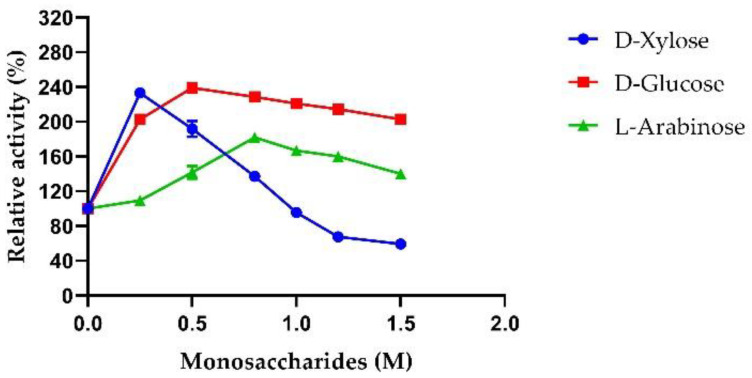
Effect of monosaccharides on AmβXyl activity. D-xylose (circles), D-glucose (squares), and L-arabinose (triangles).

Therefore, the monosaccharide tolerance of AmβXyl was investigated. Our results showed that AmβXyl retained 100% of its activity in the presence of 1 M xylose and 60% in the presence of 1.5 M xylose. In addition, an increased activity of ~2.5-fold was observed in the presence of 0.25 M xylose (Figure 9). On the other hand, the presence of glucose and arabinose in a range of 0.5–1.5 M stimulated AmβXyl activity; maximal increases in the enzymatic activity of ~1.8-fold in the presence of 0.8 M arabinose and ~2.4-fold in the presence of 0.5 M glucose were observed (Figure 9).

## 3. Materials and Methods

### 3.1. Gene Synthesis

*E. coli* strains were grown in solid or liquid LB medium at 37 °C containing tetracycline (15 μg/μL) and kanamycin (50 μg/μL) (strain TopF’10) or chloramphenicol (33 μg/μL) and kanamycin (50 μg/μL) (strain BL21 (DE3) RIL). AmβXyl gene (locus tag IW967_11185) encoding the putative β-xylosidase from *A. mali* FL18 (GenBank Accession No. JADPKZ000000000) was synthesized by GenScript Biotech (Piscataway, NJ, USA), by optimizing the codon usage for the expression in *E. coli*. The optimized gene was cloned in pET-30a(+) vector (pET30a-AmβXyl), by using *Nco*I and *Hind*III as restriction sites, with an in-frame His-tag at the N-terminus.

### 3.2. Sequence Analyses of AmβXyl 

The genome of *A*. *mali* FL18 was previously sequenced and annotated with NCBI and RAST [21]. Among several GHs retrieved in the genome, a β-xylosidase annotated as GH3 was selected. The protein sequence was analyzed by BlastP (www.Blast.ncbi.nlm.nih.gov/Blast.cgi, accessed on 15 May 2022) and CAZy (www.Cazy.org, accessed on 15 May 2022), and the possible presence of a signal sequence and N-glycosylation sites was analyzed using the tools SignalP 6.0 (https://services.healthtech.dtu.dk/service.php?SignalP, accessed on 15 May 2022) and NetNGlyc (https://services.healthtech.dtu.dk/service.php?NetNGlyc-1.0, accessed on 15 May 2022). The Pfam-*(EMBL-EBI)* (http://pfam.xfam.org/search/sequence, accessed on 15 May 2022) and I-TASSER (http://zhanglab.ccmb.med.umich.edu/I-TASSER/, accessed on 15 May 2022) tools were used to analyze the structural model, the predicted catalytic sites, and the presence of conserved domains in AmβXyl sequence. The amino-acid sequence was also aligned using the CLC Main Workbench 22.0.1 multiple sequence alignment tool with other GH3 β-xylosidases from *Thermotoga petrophila* (ABQ46867.1)*, Pseudothermotoga thermarum* (AEH50242.1), *Dictyoglomus turgidum* (ACK42133.1)*, D. thermophilum* (WP012548714.1), and *Talaromyces amestolkiae* (KP119719.1). On the basis of the alignment, phylogenetic relationships were deduced using the neighbor-joining (NJ) method and the Jukes–Cantor protein distance measure. A total of 200 bootstrap replicates were used for evaluating the tree’s topological structure.

### 3.3. Expression and Purification of AmβXyl in E. coli

The *E*. *coli* BL21 (DE3) RIL strain was transformed with the vector pET30a-AmβXyl to express the recombinant protein. The clone was grown in LB containing kanamycin 50 μg/mL and chloramphenicol 33 μg/mL at 37 °C (180 rpm/min in an orbital shaker) until the optical density at 600 nm was 0.5. Protein expression was induced by adding 0.5 mM isopropyl-β-D-1-thiogalactopyranoside (IPTG) for 16 h at 37 °C. The bacterial culture (1L) was harvested by centrifugation (5000× *g*, 15 min, 4 °C); then, the pellet was resuspended in 30 mL of buffer A (20 mM imidazole, 100 mM NaCl, 50 mM sodium phosphate pH 7.5, and 1 mM DTT) supplemented with a protease inhibitor cocktail (Sigma-Aldrich, St. Louis, MO, USA) and underwent sonication (10 s of pulse on and 10 s of pulse off) for 10 min (Sonicator Heat System Ultrasonic, Inc., Farmingdale, NY, USA). The crude extract was centrifugated at 40,000× *g* for 30 min at 4 °C, to remove protein aggregates. The supernatant, containing AmβXyl, was purified by a first step of affinity chromatography (HisTrapHP 1 mL column, GE Healthcare, Chicago, IL, USA), connected to an AKTA system and equilibrated in buffer A. The protein elution was carried out with a linear gradient of imidazole (0–500 mM), and all the fractions were pooled and dialyzed against 50 mM sodium phosphate pH 7.5 and 1 mM DTT at 4 °C for 16 h. Subsequently, the extract was further purified by anionic exchange chromatography on a HiTrap Q HP column (GE Healthcare, 5 mL). The column was equilibrated in buffer A (50 mM sodium phosphate and 1 mM DTT), and elution was performed through a linear gradient from 0 to 1 M NaCl. The fractions were dialyzed against 100 mM NaCl, 50 mM sodium phosphate pH 7.5, and 1 mM DTT, and then concentrated using an Amicon Ultrafiltration System (Millipore, Burlington, MA, USA) with a 10 kDa cutoff nitrocellulose membrane (Millipore) at room temperature and a maximum pressure of 75 MPa. The homogeneity of the protein was assessed by 12% SDS-PAGE stained with Coomassie brilliant blue R-250. Lastly, the purified protein concentration was estimated using the Bradford assay with bovine serum albumin as a standard.

### 3.4. AmβXyl Biochemical Characterization

#### 3.4.1. Quaternary Structure Determination

The native molecular weight of the purified AmβXyl was analyzed using a size-exclusion chromatograph connected to a Mini DAWN Treos light scattering system (Wyatt Technology) equipped with a QELS (quasi-elastic light scattering) module mass value and hydrodynamic radius (*R*h) measurements [52]. Briefly, 500 μg of sample was loaded on an S200 column (16/60, GE Healthcare), equilibrated in 100 mM NaCl, 25 mM sodium phosphate pH 7.5, and 1 mM DTT. A constant flow rate of 0.5 mL/min was applied. Data were analyzed using Astra 5.3.4.14 software (Wyatt Technology, Santa Barbara, CA, USA).

#### 3.4.2. β-Xylosidase Activity

The catalytic activity of the purified β-xylosidase was determined using *para*-nitrophenyl-β-D-xylopyranoside (PNP-β-xyl) as substrate. The reaction mixture containing 4 mM PNP-β-xyl, 50 mM sodium citrate (pH 5.6), and 1 mM DTT in a volume of 158 μL was preincubated at 80 °C for 2 min before the addition of 2 μL of purified enzyme (0.05 μg/μL). The reaction was carried out for 1 min at 80 °C and stopped by adding 160 μL of cold 0.5 M Na_2_CO_3_. All enzymatic reactions were carried out in triplicate into 1.5 mL tubes and then transferred to a 96-well microplate. The concentration of released *para*-nitrophenol (*p*NP) (ε_mM_, 18.5 mM^−1^·cm^−1^) was immediately detected by measuring A_405nm_ with a microplate reader (Synergy H4 Biotek, Agilent, Santa Clara, CA, USA). One unit of β-xylosidase was defined as the amount of enzyme required to release 1 μmol of *p*NP per min under assay conditions.

#### 3.4.3. Substrate Specificity and Kinetic Parameters

The substrate specificity of AmβXyl was tested firstly using the following substrates: *para*-nitrophenyl-β-D-xylopyranoside (PNP-β-xyl), *para*-nitrophenyl-α-L-arabinofuranoside (PNP-α-Ara) *para*-nitrophenyl-α-D-galactopyranoside (PNP-α-gal), *para*-nitrophenyl-β-D-galactopyranoside (PNP-β-gal), *para*-nitrophenyl-α-D-glucopyranoside (PNP-α-glu), *para*-nitrophenyl-β-D-glucopyranoside (PNP-β-glu)*, para*-nitrophenyl-α-D-glucopyranoside (PNP-α-glu), beechwood xylan, and wheat arabinoxylan. All enzymatic measurements were performed in triplicate. The concentration of released *p*NP was determined by measuring A_405nm_, while xylanase and arabinoxylanase activities were determined with the 3,5-dinitrosalicylic acid (DNS) method [53]. One unit of xylanase or arabinoxylanase activity was defined as the amount of enzyme required to release 1 μmol of reducing sugar equivalent to xylose or arabinose from beechwood xylan or wheat arabinoxylan per minute under standard assay conditions.

Kinetic parameters, K_M_, V_max_, *k*_cat_, and *k*_cat_/K_M,_ were determined through the standard assay procedure utilizing PNP-β-xyl and PNP-α-ara as substrates at concentrations ranging from 0.1 to 8.0 mM using GraphPad 8.0 Prism software.

#### 3.4.4. TLC Analysis

Xylose (X), xylobiose (X_2_), and xylotriose (X_3_) were purchased from Biosinth S.R.O. XOS were treated with purified AmβXyl, and hydrolysis products were analyzed by thin-layer chromatography (TLC). The reaction mixture (50 μL) contained 10 mM XOS and 0.2U AmβXyl in 50 mM sodium citrate (pH 5.6) and 1 mM DTT. Incubation times of 1 min, 30 min, and 2 h were tested at 80 °C and stopped by exposure to dry ice for 10 min. The reaction mixtures (12.5 μL) were loaded on silica gel 60 (F254, 0.25 mm) plates (Merck, Darmstadt, Germany) and separated using n-butanol/ethanol/ddH_2_O (2:1:1 *v*/*v*/*v*) as the eluent. For the detection of sugars, the plates were soaked in a solution consisting of ethanol/sulfuric acid (90:10 *v*/*v*), followed by heating for few minutes at 120 °C in an oven. Mixture without enzyme was included in the analysis as control.

#### 3.4.5. pH and Temperature Effects on Activity and Stability of the Enzyme

The optimal pH of recombinant AmβXyl was evaluated by performing the β-xylosidase assay using PNP-β-xyl as a substrate, as reported above, in the pH range 3.0–8.0, at the temperature reported above.

To determine enzyme stability at different pH values, assays were carried out by incubating AmβXyl in buffers ranging from pH 3.0 to 8.0 at 4 °C for 16 h. The residual β-xylosidase activity was measured under the standard conditions as previously reported.

The optimal temperature was determined by measuring the β-xylosidase activity at different temperatures ranging from 30 to 90 °C. Thermal stability was determined through preincubation of the enzyme at different temperatures (40, 50, 60, 65, 70, and 80 °C) in the reaction mixture for different times (10–120 min) without substrate. Aliquots of AmβXyl were taken at regular time intervals to measure the residual activity under standard assay conditions. The activity of the enzyme assayed without preincubation was defined as 100%.

#### 3.4.6. Effect of Chemicals and Monosaccharides on Enzyme Activity

The inhibition effect of metal ions and chelating agent EDTA on AmβXyl activity was evaluated. The effect of different ions Cu^2+^, Zn^2+^, Li^+^ Mg^2+^, Ca^2+^, Mn^2+^, and Ni^2+^, as well as the chemical agent EDTA, at final concentrations of 1 mM and 5 mM on enzymatic activity was determined.

Enzymatic activity was measured in the presence of nonionic (Tween-20 and Triton X-100) and ionic (SDS) detergents at a final concentration of 0.5% (*v*/*v*).

The β-xylosidase activity was also detected in the presence of different concentrations of D-xylose, D-glucose, L-arabinose (0.5–1.5 M), and NaCl (0.25–2.5 M) under standard assay conditions.

Lastly, AmβXyl activity was tested at 60 °C and optimal pH with the addition of organic solvents (DMSO, ethanol, or methanol) at final concentrations of 10%, 20%, 30%, 40%, 50%, and 60% in the reaction mixture. The activity of the enzyme without organic solvents assayed at 60 °C was defined as 100%.

## 4. Conclusions

In this study, a thermophilic GH3 β-xylosidase (AmβXyl) from the thermoacidophile bacterium *A. mali* FL18 was biochemically characterized. AmβXyl displays a peculiar thermophilicity, thermoresistance, and stability in a wide range of pH. A common characteristic of several enzymes of the GH3 family is bifunctionality; in particular, AmβXyl displays a good catalytic efficiency on both PNP-β-xyl and PNP-α-ara synthetic substrates. Furthermore, AmβXyl is capable of hydrolyzing XOS, playing an important role in the saccharification process since it relieves the end-product inhibition of endoxylanase. Moreover, AmβXyl has shown an excellent tolerance to organic solvents making it a suitable enzyme for several biotechnological applications. Interestingly, AmβXyl is the first β-xylosidase reported to be not only resistant to end-product inhibition (i.e., xylose and arabinose) but also stimulated in the presence of high concentrations of several monosaccharides. Altogether, the data presented in this study are a good starting point both for the formulation of novel enzymatic mixtures and for the valorization of hemicellulosic materials in the context of a green economy.

## Figures and Tables

**Table 1:** Purification steps of AmβXyl. Abbreviations: C.E., culture extract; A.C., affinity chromatography; A.E.C., anionic exchange chromatography.

Purification Step	Total Activity (U)	Total Protein (mg)	Specific Activity (U/mg)	Yield (%)	Fold Purification
C.E.	6300.00	281.29	22.39	100.00	1.00
A.C.	1470.60	3.87	380.00	23.30	16.97
A.E.C.	756.00	1.26	600.00	12.00	26.80

**Table 2:** Substrate specificity of AmβXyl. ND: non detected.

Substrate	Relative Activity (%)
PNP-β-xyl	100
PNP-α-ara	59.30
PNP-β-glu	0.19
PNP-α-glu	0.73
PNP-β-gal	ND
PNP-α-gal	ND
Beechwood xylan	14.59
Wheat arabinoxylan	20.86

**Table 3:** Comparison of kinetic parameters of AmβXyl with those of some thermophilic β-xylosidases. ND: non detected.

Substrate	Enzyme	K_M_ (mM)	*k*_cat_ (s^−1^)	*k*_cat_/K_M_ (mM^−1^·s^−1^)	Reference
PNP-β-xyl	Am * βXyl *	0.52	1606.00	3088.46	This work
	TthxynB3	0.27	316.81	1173.40	[29]
	Dt-Xyl3	0.83	ND	ND	[28]
	Tpe-Xyl3	0.12	982.32	7808.00	[30]
	Dt-2286	0.12	651.59	5245.31	[27]
PNP-α-ara	Am * βXyl *	10.56	2395.80	226.87	This work
	TthxynB3	0.21	106.23	505.90	[29]
	Dt-Xyl3	2.01	ND	ND	[28]
	Tpe-Xyl3	6.16	2201.64	357.89	[30]
	Dt-2286	0.50	1054.33	2077.35	[27]

**Table 4:** Effect of metal ions and chemicals on AmβXyl activity. ND: non detected.

Metal Ions or Chemical Agents		Relative Activity (%)	
	1 mM	5 mM	0.5%
None	100	100	100
CuCl_2_	32.10	ND	
ZnCl_2_	105.80	93.99	
LiCl	89.85	83.20	
MgCl_2_	91.64	77.81	
CaCl_2_	82.97	82.46	
MnSO_4_	78.13	59.59	
NiCl_2_	104.28	86.29	
EDTA	96.99	98.89	
SDS			1.30
Triton X-100			91.08
Tween 20			113.36
